# Sex and age differences in the patient-reported outcome measures and adherence to an osteoarthritis digital self-management intervention

**DOI:** 10.1016/j.ocarto.2024.100437

**Published:** 2024-01-28

**Authors:** Simone Battista, L Stefan Lohmander, Andrea Dell’Isola, Leif E. Dahlberg, Ali Kiadaliri

**Affiliations:** a Clinical Epidemiology Unit, Department of Clinical Sciences Lund, Orthopaedics, Lund University, Lund, Sweden; b Department of Neurosciences, Rehabilitation, Ophthalmology, Genetics, Maternal and Child Health, University of Genova, Campus of Savona, Italy; c Department of Clinical Sciences Lund, Orthopaedics, Lund University, Lund, Sweden; d Arthro Therapeutics, Malmö, Sweden

**Keywords:** Digital technology, Physical therapy modalities, Healthcare disparities

## Abstract

**Objective:**

To explore sex and age differences in Patient-Reported Outcomes Measures (PROMs) and adherence to digital osteoarthritis (OA) self-management intervention.

**Methods:**

A register-based study with data from an OA digital self-management intervention. PROMs and adherence were collected at baseline and/or 3 ​month follow-up: ‘pain intensity’ in hip/knee (best/worst: 0–10), ‘activity impairments' (best/worst: 0–10), ‘overall health’ perception (worst/best: 0–10), ‘physical function’ (30-s chair stand test), ‘health-related quality of life’ (EQ-5D-5L index score; worst/best: 0.243–0.976), the subscales and total scores of the Knee Injury/Hip Disability and Osteoarthritis Outcome Score (KOOS/HOOS-12; worst/best: 0–100), ‘fear of movement’ (yes/no), ‘walking difficulties' (yes/no), ‘programme adherence’ (0–100 ​% and ≥80 ​% [yes/no]), ‘patient acceptable symptom state’ (PASS; yes/no), and ‘treatment failure’ (those who answered no to PASS question and thought the treatment failed [yes/no]). We used linear/logistic regression to calculate mean/risk differences in the PROMs and adherence levels among sex and age groups at 3-month follow-up. We employed entropy balancing to explore the contributions of baseline characteristics and different covariates to the sex/age differences.

**Results:**

We included 14,610 participants (mean (SD) age: 64.1 (9.1), 75.5 ​% females). Females generally reported better outcomes than males. Participants aged ≥70 had greater activity impairments, lower KOOS/HOOS-pain/function scores, more walking difficulties, less fear of movement and higher adherence than those <70. However, these differences were small and not likely clinically relevant.

**Conclusion:**

No clinically relevant differences in PROMs and adherence were found among sex/age groups in this digital OA programme, suggesting that sex/age seemed not to impact the outcomes of this intervention.

## 1 Introduction

OA care focusses on symptom management with exercise and education as first-line interventions [1]. Different face-to-face or digital OA self-management interventions based on exercise and education have been implemented to increase the administration of and adherence to these interventions [2,3], suggesting comparable outcomes between face-to-face and digital modalities [4].

In 2022, the European Alliance of Associations for Rheumatology (EULAR) developed its ‘Points to consider for remote care in rheumatic and musculoskeletal diseases’, underlining the importance of evaluating and resolving any possible barriers hindering digital intervention use and efficacy [5]. A recent scoping review mapped the inequities of digital health technologies (access, use and engagement) within the World Health Organisation's European region, indicating greater use of and access to digital technologies among women and young adults [6]. Disparities in OA first-line interventions have been highlighted [7]. Still, there has been limited consideration of potential sex/age disparities in the outcomes of individuals who are enrolled in a digital intervention, especially in OA.

A digital self-management platform for people with OA was recently introduced in Sweden. Past evidence on this platform showed that the female sex was generally positively associated with adherence [3]. Moreover, age and adherence showed a nonlinear relationship where a 1-year increase in age was associated with a 0.3 ​% increase in 3-month adherence up to age 75 and a 0.5 ​% reduction thereafter (age >75) [3]. Nevertheless, age and sex could only explain a small percentage of adherence variance [3]. Improvements in both activity and work impairments for females and younger adults differed positively from those of males and older adults; however, these differences were not statistically conclusive [8]. None of these studies explored sex/age differences in Patient-Reported Outcome Measures (PROMs) beyond work/activity impairments (e.g., pain intensity, fear of movement, etc.) and adherence [3,8]. Hence, the present study aimed to explore sex and age differences in the PROMs and adherence within an OA digital self-management intervention, focussed on exercise and education, to ascertain whether individuals with OA can benefit from such interventions regardless of sex and age once provided with similar opportunities and abilities to utilise these resources.

## 2 Methods

### 2.1 Study design and setting

We conducted a register-based study on individual-level data extracted on January 12th, 2022, from the ‘Joint Academy’ (www.jointacademy.com), a digital smartphone app-based self-management platform for people with OA [3]. The programme is a structured digital treatment for people with hip or knee OA based on exercise tailored to the participants. Generally, people attend two sessions of exercise per day. They cannot continue the programme unless they mark the sessions as completed. Education sessions are implemented within the programme, delivered asynchronously through text or video lessons and followed by quizzes on the materials. Each lesson is flagged as done once participants complete the quiz correctly. The overall programme spans 48 ​weeks. However, the core content and basic package are delivered within 12 ​weeks (3 ​months). Participants who choose to continue beyond this period receive supplementary materials and exercises. Across the recommended 12-week timeframe, representing the minimum recommended duration for a self-management intervention targeting OA, the app provides users 163 exercise sessions and 31 educational sessions [9]. Finally, participants can contact their assigned physiotherapist through an encrypted chat function and telephone. This digital intervention is reimbursed by the Swedish National Healthcare System. Participants in the programme provide digital informed consent for data collection, analysis and publication. The research was conducted with respect to the Declaration of Helsinki and reported following the Strengthening the Reporting of Observational studies in Epidemiology (STROBE). Ethical approval was obtained from the Swedish Ethical Review Authority (Dnr: 2018/650. 2019–02232 and 2020–05431).

### 2.2 Participants

We included all participants with hip or knee OA enrolled in the digital programme between January 1st, 2019 and September 30th, 2021, who provided informed consent for research (*n* ​= ​16,640). We excluded 2030 (12.2 ​%) who did not respond to the 3-month follow-up.

### 2.3 Outcomes

Participants recorded their PROMs following the instructions on the digital platform at both baseline and 3-month follow-up: ‘pain intensity’ in the ‘index joint’ (numeric rating scale [NRS] 0–10 with higher score indicating higher pain); ‘activity impairments’ from the ‘Work Productivity and Activity Impairment’ questionnaire (NRS 0–10, “During the past seven days how much did OA affect your ability to do your regular daily activities, other than work at a job?” with higher score indicating higher impairment); ‘overall health’ (NRS 0–10, “Mark on the scale how good or bad your current health is?” with 0 ​= ​worst imaginable and 10 ​= ​best imaginable); ‘physical function’ was assessed with the 30-s chair stand test (30 CST) performed by the participants following an instruction video with a digital stopwatch (with higher values indicating better performance) [10]; ‘European Quality of Life 5 Dimensions 5 Level Version (EQ-5D-5L) index score’ to measure participants' health-related quality of life (Swedish value set ranging from 0.243 to 0.976); the subscales (Pain, Function, Quality of Life) and total scores of the 12-item short forms of the ‘Knee injury and Osteoarthritis Outcome Score’ (KOOS-12) and ‘Hip disability and Osteoarthritis Outcome Score’ (HOOS-12) (both ranged 0–100 with higher score indicating better outcomes); ‘fear of movement’ (“Are you afraid your joints will be injured by physical training/activity?” ‘yes/no’); and ‘walking difficulties’ (“Do you have problems walking as a result of your joint problems?” ‘yes/no’). Adherence was measured at 3-month follow-up: ‘adherence’ defined as the percentage (0–100) of completed activities (exercises, text or video lessons, and quizzes on lesson material) over 12-weeks (12-week average) [3]; ‘desirable adherence’ was defined as adherence ≥80 ​%’ (yes/no). These PROMs were measured only at 3 ​months: ‘Patient Acceptable Symptom State’ (PASS) (yes/no answer to the question “Considering your hip/knee function, do you feel that your current state is satisfactory? With hip/knee function, you should take into account all activities you have during your daily life, sport and recreational activities, your level of pain and other symptoms, and quality of life”); and ‘Treatment Failure’ (those who answered no to PASS question were asked “Would you consider your current state as being so unsatisfactory that you think the treatment has failed?’’ with yes/no answers). Those who answered yes to PASS were categorised as ‘no’ treatment failure.

### 2.4 Exposure variables

The participants' ‘assigned sex at birth’ (female/male) and ‘age’ (<60 ​years/60–69 ​years/70+ ​years) were the primary exposures.

### 2.5 Covariates

The following variables were also measured and included as possible factors contributing to any sex/age differences in the outcomes: ‘index joint’ (i.e. most painful joint with OA as reported by participants categorised as hip or knee); ‘educational attainment’ (less than high school/high school/college or university); ‘employment’ (working/not working/retired); ‘height and weight’’; the presence of coexisting conditions (diabetes/lung diseases/balance troubles/rheumatoid arthritis/cardiovascular diseases/pain in other joints)’ with yes/no answers; and the ‘year of enrolment’ (2019/2020/2021).

### 2.6 Statistical analysis

Descriptive statistics are reported as mean, standard deviation (SD), and absolute and percentage frequencies. We computed standardised mean differences (SMDs) using the ‘stddiff’ Stata command for descriptive and outcome variables between those included and excluded from the analysis identifying group imbalances where SMD >0.1. Then, to investigate the sex and age gaps in the outcomes at a 3-month follow-up, we performed a three-step analysis. Firstly, we computed crude differences in the outcomes of interest among sex/age groups. Secondly, we used entropy balancing (Stata command ‘ebalance’) to adjust for the differences in the baseline values of the outcomes of interest across sex/age groups. Entropy balancing is a pre-processing, multivariate reweighting method to achieve covariate balance across two groups [11]. In other words, the sample in one group is weighted to have a similar first, second, and higher moments of the covariate distribution as the sample in the other group. Thirdly, we included all covariates mentioned above and the baseline value of the outcomes of interest in entropy balancing. In this study, we used entropy balancing to achieve balance in mean, variance and skewness of covariate(s) across groups. Estimates from steps 2 and 3 for an outcome can be interpreted as the differences in the 3-month outcome across sex/age groups that cannot be explained by differences in baseline outcome (step 2) and covariates (step 3). We used linear regression for NRS and continuous outcomes to compute mean difference and logistic regression and Stata ‘margins’ command for binary outcomes to compute risk differences. For outcomes only measured at 3-month follow-up (i.e. adherence, PASS, and treatment failure), we skipped step 2 and only balanced groups for covariates. Due to low variation in employment status among those aged 70+ ​years (i.e. most people in this age group were retired), we did not include ‘employment’ as a covariate when analysing age gaps in the outcomes. The analysis was done by Stata 17 (College Station, TX: StataCorp LLC).

## 3 Results

A total of 14,610 participants (mean (SD) age: 64.1 (9.1), 75.5 ​% females) were included in the study. Among them, 10,402 (71.2 ​%) answered all the outcomes under study, and 3181 (21.8 ​%) responded to all but physical function tests. Table 1 reports the descriptive statistics and the baseline outcomes stratified by sex and age categories.

**Table 1:** Participants’ baseline characteristics and outcomes stratified by sex and age.

	Sex groups		Age groups		
	Females	Males	<60 ​years	60–69 ​years	70+ ​years
***N***	11,029	3581	4346	5988	4276
**Females, *n* (%)**	–	–	3341 (76.9)	4631 (77.3)	3057 (71.5)
**Age, mean (SD)**	63.8 (8.9)	64.8 (9.5)	53.1 (5.1)	64.6 (2.8)	74.4 (3.7)
**Index joint, *n* (%)**					
Knee	6497 (58.9)	2261 (63.1)	2617 (60.2)	3652 (61.0)	2489 (58.2)
Hip	4532 (41.1)	1320 (36.9)	1729 (39.8)	2336 (39.0)	1787 (41.8)
**Educational attainment, *n* (%)**					
Less than high school	848 (7.7)	333 (9.3)	161 (3.7)	444 (7.4)	576 (13.5)
High school	3759 (34.1)	1485 (41.5)	1841 (42.4)	2242 (37.4)	1161 (27.2)
College/university	6422 (58.2)	1763 (49.2)	2344 (53.9)	3302 (55.1)	2539 (59.4)
**Employment, *n* (%)**					
Working	4876 (44.2)	1570 (43.8)	3807 (87.6)	2504 (41.8)	135 (3.2)
Not working	602 (5.5)	113 (3.2)	434 (10.0)	274 (4.6)	7 (0.2)
Retired	5551 (50.3)	1898 (53.0)	105 (2.4)	3210 (53.6)	4134 (96.7)
**Body mass index, mean (SD)**	27.2 (5.0)	27.3 (3.9)	28.1 (5.4)	27.2 (4.5)	26.3 (4.0)
**Coexisting conditions, *n* (%)**					
Diabetes	534 (4.8)	285 (8.0)	145 (3.3)	338 (5.6)	336 (7.9)
Lung diseases	1298 (11.8)	258 (7.2)	447 (10.3)	604 (10.1)	505 (11.8)
Balance troubles	369 (3.4)	114 (3.2)	90 (2.1)	165 (2.8)	228 (5.3)
Rheumatoid arthritis	526 (4.8)	136 (3.8)	140 (3.2)	270 (4.5)	252 (5.9)
Cardiovascular diseases	632 (5.7)	446 (12.5)	111 (2.6)	385 (6.4)	582 (13.6)
Pain other joints	8817 (79.9)	2398 (67.0)	3327 (76.6)	4688 (78.3)	3200 (74.8)
**Year of enrolment, *n* (%)**					
2019	720 (6.5)	205 (5.7)	285 (6.6)	387 (6.5)	253 (5.9)
2020	2769 (25.1)	897 (25.1)	1100 (25.3)	1514 (25.3)	1052 (24.6)
2021	7540 (68.4)	2479 (69.2)	2961 (68.1)	4087 (68.3)	2971 (69.5)
**Pain (NRS 0**–**10), mean (SD)**	5.1 (1.9)	4.9 (1.9)	5.3 (2.0)	5.1 (1.9)	4.9 (1.9)
**Activity Impairment (NRS 0**–**10), mean (SD)**	3.9 (2.4)	3.9 (2.3)	4.1 (2.5)	3.9 (2.4)	3.9 (2.3)
**Overall Health (NRS 0**–**10), mean (SD)**	6.5 (1.9)	6.8 (1.8)	6.3 (1.9)	6.6 (1.8)	6.8 (1.8)
**Physical function, mean (SD)**	12.5 (4.1)	13.6 (4.9)	13.3 (4.6)	12.7 (4.2)	12.3 (4.2)
**EQ-5D-5L index score, mean (SD)**	0.82 (0.11)	0.82 (0.11)	0.81 (0.12)	0.83 (0.10)	0.83 (0.11)
**KOOS/HOOS-PAIN, mean (SD)**	52.2 (16.6)	54.5 (15.5)	51.7 (17.2)	53.0 (16.4)	53.5 (15.4)
**KOOS/HOOS-FUNCTION, mean (SD)**	61.6 (19.3)	64.6 (18.4)	61.8 (20.2)	62.6 (19.0)	62.6 (18.3)
**KOOS/HOOS-QoL, mean (SD)**	45.9 (17.3)	45.9 (16.5)	43.9 (17.6)	46.3 (16.9)	47.3 (16.6)
**KOOS/HOOS-TOTAL, mean (SD)**	53.2 (15.6)	55.0 (14.6)	52.5 (16.1)	54.0 (15.3)	54.5 (14.6)
**Fear of movement, *n* (%)**	1548 (14.0)	657 (18.4)	1122 (25.8)	762 (12.7)	321 (7.5)
**Walking difficulties, *n* (%)**	7188 (65.2)	2397 (66.9)	2831 (65.1)	3907 (65.3)	2847 (66.6)

N, number; SD, standard deviation.

Knee OA was more prevalent than hip OA across the sex/age groups. Except for the variable ‘fear of movement’, the baseline outcomes were generally comparable across the sex/age groups. The baseline characteristics of the included participants were mostly comparable to those excluded, with some exceptions noted in age, knee as the index joint, employment status, presence of diabetes, balance troubles, cardiovascular diseases, physical function, and EQ-5D-5L (SMD >0.1, see Supplementary File 1).

Table 2 illustrates the disparities in the outcomes at the 3-month follow-up across age and sex groups before and after achieving balance. After adjustment for baseline outcomes and covariates through balancing, females reported better outcomes for pain, EQ-5D-5L, KOOS-12/HOOS-12, fear of movement, walking difficulties, PASS and treatment failure. At the same time, physical function was better among males. Across age groups, participants aged ≥70 had greater activity impairments and walking difficulties, lower KOOS/HOOS-pain and function scores, less fear of movement and higher adherence than those aged <70. However, these sex and age differences were predominantly statistical, mostly small in magnitude, and likely not clinically relevant.

**Table 2:** Differences in the outcomes at 3-month follow-up between age and sex groups.

	Differences at 3-month follow-up [95 ​% CI]								
	≥70 vs. <60 ​years			≥70 vs. 60–69 ​years			Females vs. males		
	Crude	Baseline outcome balance	Baseline outcome ​+ ​covariates balance	crude	Baseline outcome balance	Baseline outcome ​+ ​covariates balance	Crude	Baseline outcome balance	Baseline outcome ​+ ​covariates balance
Pain (NRS 0–10) MD [95 ​% CI]	−0.16 [−0.25; −0.07]	0.03 [−0.06; 0.11]	−0.02 [−0.15; 0.11]	0.06 [−0.02; 0.14]	0.12 [0.04; 0.20]	0.13 [0.05; 0.22]	0.05 [−0.02; 0.13]	−0.07 [−0.015; 0.01]	−0.12 [−0.21; −0.03]
Activity impairments (NRS 0–10) MD [95 ​% CI]	0.00 [−0.09; 0.10]	0.08 [−0.01; 0.18]	0.14 [−0.01; 0.29]	0.15 [0.06; 0.24 ]	0.12 [0.03; 0.21]	0.21 [0.12; 0.30]	−0.01 [−0.09; 0.08]	−0.02 [−0.11; 0.06]	−0.09 [−0.19; 0.02]
Overall health (NRS 0–10) MD [95 ​% CI]	0.42 [0.34; 0.51]	0.19 [0.11; 0.28]	0.22 [0.10; 0.35]	0.08 [−0.0004; 0.15]	0.01 [−0.08; 0.08]	−0.04 [−0.09; 0.08]	−0.23 [−0.30; −0.16]	−0.12 [−0.19; −0.05]	−0.07 [−0.16; 0.01]
Physical function (s) MD [95 ​% CI]	−2.5 [−2.8; −2.3]	−1.7 [−2.0; −1.4]	−1.7 [−2.2; −1.2]	−1.4 [−1.6; −1.1]	−1.1 [−1.3; −0.8]	−1.3 [−1.5; −1.0]	−1.6 [−1.9; −1.4]	−0.7 [−0.9; −0.4]	−0.9 [−1.2; −0.6]
EQ-5D-5L index score (0.243–0.976) MD [95 ​% CI]	0.017 [0.013; 0.022]	0.000 [−0.004; 0.005]	0.010 [0.002; 0.017]	0.000 [−0.004; 0.004]	−0.003 [−0.007; 0.001]	−0.003 [−0.007; 0.002]	−0.001 [−0.006; 0.003]	−0.002 [−0.004; 0.004]	0.009 [0.003; 0.014]
KOOS/HOOS-PAIN (0–100) MD [95 ​% CI]	−0.1 [−0.9; 0.7]	−1.3 [−2.0; −0.5]	−1.2 [−2.4; −0.02]	−0.8 [−1.5; −0.1]	−1.1 [−1.8; −0.4]	−1.4 [−2.1; −0.6]	−0.5 [−1.2; 0.2]	0.9 [0.2; 1.6]	0.9 [0.04; 1.7]
KOOS/HOOS-FUNCTION (0–100) MD [95 ​% CI]	−1.2 [−2.0; −0.3]	−1.8 [−2.6; −0.9]	−2.4 [−3.7; −1.1]	−1.0 [−1.8; −0.2]	−1.0 [−1.8; −0.3]	−1.5 [−2.3; −0.7]	−1.3 [−2.1; −0.6]	0.5 [−0.3; 1.3]	0.2 [−0.7; 1.1]
KOOS/HOOS-QoL (0–100) MD [95 ​% CI]	3.2 [2.3; 4.0]	0.6 [−0.2; 1.4]	1.4 [0.2; 2.6]	0.5 [−0.2; 1.2]	−0.2 [−0.9; 0.5]	−0.2 [−1.0; 0.6]	1.0 [0.3; 1.7]	1.0 [0.2; 1.7]	2.0 [1.2; 2.9]
KOOS/HOOS-TOTAL (0–100) MD [95 ​% CI]	0.6 [−0.1; 1.4]	−0.9 [−1.6; −0.2]	−0.7 [−1.8; 0.4]	−0.4 [−1.1; 0.2]	−0.8 [−1.4; −0.1]	−1.0 [−1.8; −0.3]	−0.3 [−0.9; 0.4]	1.0 [0.3; 1.7]	1.0 [0.2; 1.8]
Fear of movement (yes/no) RD [95 ​% CI]	−8.7 [−9.8; −7.6]	−3.4 [−4.2; −2.6]	−3.9 [−5.2; −2.7]	−2.3 [−3.1; −1.6]	−1.2 [−1.9; −0.6]	−1.1 [−1.8; −0.4]	−1.6 [−2.6; −0.6]	−0.6 [−1.5; 0.3]	−1.3 [−2.4; −0.2]
Walking difficulties (yes/no) RD [95 ​% CI]	3.2 [1.1; 5.3]	2.5 [0.4; 4.6]	3.3 [0.2; 6.4]	2.4 [0.4; 4.3]	1.7 [−0.3; 3.6]	2.3 [0.2; 4.4]	−1.8 [−3.7; 0.02]	−0.9 [−2.8; 0.9]	−2.2 [−4.5; −0.01]
Adherence (0–100 ​%) MD [95 ​% CI]	5.5 [4.8; 6.2]	N.A.	5.2 [4.2; 6.3]	0.9 [0.3; 1.5]	N.A.	0.7 [0.1; 1.4]	0.9 [0.3; 1.5]	N.A.	0.7 [−0.04; 1.4]
Adherence ​≥ ​80 ​% RD [95 ​% CI]	13.4 [11.6; 15.3]	N.A.	11.5 [8.7; 14.2]	2.4 [0.9; 4.0]	N.A.	1.9 [0.2; 3.7]	2.0 [0.3; 3.6]	N.A.	1.4 [−0.6; 3.3]
PASS (yes/no) RD [95 ​% CI]	8.2 [6.1; 10.2]	N.A.	4.4 [1.1; 7.6]	1.3 [−0.7; 3.2]	N.A.	1.0 [−1.1; 3.1]	3.1 [1.3; 5.0]	N.A.	4.6 [2.4; 6.8]
Treatment failure (yes/no) RD [95 ​% CI]	−1.6 [−2.5; −0.8]	N.A.	−1.0 [−2.1; −0.01]	−0.4 [−1.2; 0.3]	N.A.	−0.4 [−1.2; 0.4]	−1.7 [−2.5; −0.8]	N.A.	−1.9 [−2.9; −0.8]

CI, confidence interval; NRS, numeric rating scale; MD, mean difference; s, second(s); EQ-5D-5L, European Quality of Life 5 Dimensions 5 Level Version; KOOS, Knee injury and Osteoarthritis Outcome Score; HOOS, Hip disability and Osteoarthritis Outcome Score; QoL, Quality of Life; RD, risk difference; PASS, Patient Acceptable Symptom Scale; N.A., Not Available (no measure at the baseline).

## 4 Discussion

In this register-based study using data from a digital OA self-management platform, we did not find clinically relevant sex/age differences in participants’ PROMs and adherence levels.

Past evidence has suggested disparities in access to digital health technologies, with women and young adults more inclined to use digital technologies than men and older adults [12]. For women, digital technologies have shown the potential to reduce gender-related face-to-face care barriers that persist in our society, including childcare, household chores, financial constraints and time limitations [13]. Conversely, health literacy poses a significant obstacle for older adults [12]. In our present study, our focus diverged from assessing access disparities. Instead, we explored sex/age differences in PROMs and adherence within a digital self-management intervention for OA. Notably, our findings revealed no clinically important outcome disparities among age/sex groups among those who used the digital self-management intervention. Altogether, these findings contribute additional insight into previous studies on this digital intervention, where age and sex exhibited the lowest relative importance as predictors for work and activity impairment improvements, and sex was not a significant predictor of adherence [3,8].

We acknowledge that those who self-selected this OA treatment platform may have been inherently more technologically savvy than the average person with knee or hip OA, irrespective of age and sex, limiting our ability to generalise our results to the broader OA population. Second, data are self-reported and may be prone to biases. Third, this study solely focussed on elucidating variations across sex assigned at birth and age groups, and there might be unobserved factors that can explain the small differences observed in the study (e.g. gender, motivation, smoking, alcohol consumption, etc). Finally, there were some differences between those included and excluded from this study, limiting the generalisability of our results. Conversely, the large sample size, the number of covariates, the low drop-out/missingness of data, and the use of entropy balance to weigh the sex/age gaps represent the main strengths of our study.

To conclude, we did not observe any clinically relevant sex/age gaps in the PROMs and adherence to a digital self-management intervention for OA before and after adjustment for participants’ other characteristics. These findings underscore that individuals with OA can benefit from digital interventions focussed on exercise and education, regardless of sex and age, when provided with similar opportunities and abilities to utilise such interventions.

## Author contributions

All authors: (1) made substantial contributions to the conception and design, or acquisition of data, or analysis and interpretation of data; (2) participated in drafting the article or revising it critically for important intellectual content; and (3) gave final approval of the version to be published.

## Role of the funding source

There is nothing to declare.

## Declaration of competing interest

AK and LSL act as part-time advisors for Joint Academy®; LED is the founder and chief medical officer at Joint Academy®. The other authors do not have any conflict of interest to declare.
