# Supplementary material for: Sex and age differences in the patient-reported outcome measures and adherence to an osteoarthritis digital self-management intervention
Source: Osteoarthr Cartil Open. 2024 Jan 28;6(1):100437. doi: 10.1016/j.ocarto.2024.100437 (PMC10844664; doi:10.1016/j.ocarto.2024.100437)
Supplement: Multimedia component 1 [file mmc1.docx]

**Supplementary File 1** Standardised Mean Differences (SMDs) between those included and excluded in the study*

|  | **Included (n=14,610)** | **Excluded (n=2,030)** | **SMD** |
| --- | --- | --- | --- |
| **Age, mean (SD)** | 64.1 (9.1) | 65.6 (10.3) | -0.16 |
| **Female, n (%)** | 11,029 (75.5) | 1,532 (75.5) | 0.00 |
| **Knee as index joint, n (%)** | 5,852 (40.1) | 950 (46.8) | -0.14 |
| **Educational Attainment, n (%)** |  |  | 0.03 |
| Less than high school | 1,181 (8.1) | 183 (9.0) |  |
| High school | 5,244 (35.9) | 719 (35.4) |  |
| College/university | 8,185 (56.0) | 1,128 (55.6) |  |
| **Employment, n (%)** |  |  | 0.17 |
| Working | 6,446 (44.1) | 733 (36.1) |  |
| Not working | 715 (4.9) | 141 (6.9) |  |
| Retired | 7,449 (51.0) | 1,156 (56.9) |  |
| **Body mass index, mean (SD)** | 27.2 (4.7) | 27.2 (4.8) | -0.00 |
| **Coexisting conditions, n (%)** |  |  |  |
| Diabetes | 819 (5.6) | 174 (8.6) | -0.12 |
| Lung diseases | 1,556 (10.7) | 230 (11.3) | -0.02 |
| Balance troubles | 483 (3.3) | 117 (5.8) | -0.12 |
| Rheumatoid arthritis | 662 (4.5) | 135 (6.7) | -0.09 |
| Cardiovascular diseases | 1,078 (7.4) | 216 (10.6) | -0.11 |
| Pain other joints | 11,215 (76.8) | 1,575 (77.6) | -0.02 |
| **Pain (NRS, 0-10), mean (SD)** | 5.1 (1.9) | 5.2 (2.1) | -0.04 |
| **Physical function, mean (SD)** | 12.8 (4.3) | 12.0 (4.4) | 0.18 |
| **EQ-5D-5L index score, mean (SD)** | 0.823 (0.11) | 0.806 (0.13) | 0.15 |
| **KOOS/HOOS-PAIN, mean (SD)** | 53.0 (16.7) | 53.6 (17.0) | -0.04 |
| **KOOS/HOOS-FUNCTION, mean (SD)** | 61.7 (19.3) | 61.7 (20.1) | -0.00 |
| **KOOS/HOOS-QoL, mean (SD)** | 44.0 (16.7) | 44.4 (17.5) | -0.03 |
| **KOOS/HOOS-TOTAL, mean (SD)** | 52.9 (15.4) | 53.3 (15.8) | -0.02 |
| **Walking difficulties, n (%)** | 9,585 (65.6) | 1,392 (68.6) | -0.06 |
| **Fear of moving, n (%)** | 2,205 (15.1) | 295 (14.5) | 0.02 |

**Legend:** *they were excluded because they did not answer at the 3-month follow-up; N, number; SD, standard deviation.
